# *Erbb2* Is Required for Cardiac Atrial Electrical Activity during Development

**DOI:** 10.1371/journal.pone.0107041

**Published:** 2014-09-30

**Authors:** Gennadiy Tenin, Christopher Clowes, Kathryn Wolton, Eliska Krejci, Jayne A. Wright, Simon C. Lovell, David Sedmera, Kathryn E. Hentges

**Affiliations:** 1 Faculty of Life Sciences, University of Manchester, Manchester, United Kingdom; 2 Institute of Anatomy, First Faculty of Medicine, Charles University, Prague, and Institute of Physiology, Academy of Sciences of the Czech Republic, Prague, Czech Republic; 3 Syngenta Ltd, Jeallots Hill, United Kingdom; University of Houston, United States of America

## Abstract

The heart is the first organ required to function during embryonic development and is absolutely necessary for embryo survival. Cardiac activity is dependent on both the sinoatrial node (SAN), which is the pacemaker of heart's electrical activity, and the cardiac conduction system which transduces the electrical signal though the heart tissue, leading to heart muscle contractions. Defects in the development of cardiac electrical function may lead to severe heart disorders. The *Erbb2* (*Epidermal Growth Factor Receptor 2*) gene encodes a member of the EGF receptor family of receptor tyrosine kinases. The Erbb2 receptor lacks ligand-binding activity but forms heterodimers with other EGF receptors, stabilising their ligand binding and enhancing kinase-mediated activation of downstream signalling pathways. Erbb2 is absolutely necessary in normal embryonic development and homozygous mouse knock-out *Erbb2* embryos die at embryonic day (E)10.5 due to severe cardiac defects. We have isolated a mouse line, *l11Jus8*, from a random chemical mutagenesis screen, which carries a hypomorphic missense mutation in the *Erbb2* gene. Homozygous mutant embryos exhibit embryonic lethality by E12.5-13. The *l11Jus8* mutants display cardiac haemorrhage and a failure of atrial function due to defects in atrial electrical signal propagation, leading to an atrial-specific conduction block, which does not affect ventricular conduction. The *l11Jus8* mutant phenotype is distinct from those reported for *Erbb2* knockout mouse mutants. Thus, the *l11Jus8* mouse reveals a novel function of *Erbb2* during atrial conduction system development, which when disrupted causes death at mid-gestation.

## Introduction

The heart is needed to pump blood through the blood vessels by repeated, rhythmic contractions, carrying oxygen and nutrients to various parts of the body. In addition to contracting muscle cells, the adult heart requires the correct function of two systems: the sinoatrial node (SAN), which is the impulse-generating (pacemaker) tissue located in the right atrium of the heart, and the cardiac conduction system which consists of the atrioventricular node (AVN), atrioventricular bundle (AVB), bundle branches (BB) and Purkinje fibers (subendocardial branches). The cardiac conduction system transduces the electrical signals from SAN to heart muscle cells. This system consists of specialised cardiomyocytes that are able to conduct cardiac action potentials more quickly and efficiently than any other cells in the heart, allowing synchronised contractions of the atria and ventricles, and is therefore essential for maintaining a consistent heart rhythm. Developmental defects in the formation of the conduction system may lead to heart diseases such as heart block, long Q-T syndrome, atrial and ventricular fibrillation and tachycardia [1], [2].

The heart is the first functional organ to be formed during embryonic development (22 days after conception in humans, 8–8.5 days in mice) and is absolutely necessary for embryo survival and correct development. Cardiac contractions start immediately after the linear heart tube forms [3]; the sinus venosus myocardium located next to the primitive atrium acts as a primary pacemaker which later develops into the SAN [4], [5]. In contrast to the pacemaker, the cardiac conduction system develops progressively. The atrioventricular canal (AVC) at E9.5 gives rise to AVN. The AVB is formed from the crest of the developing ventricular septum; Purkinje fibers develop in the trabecular myocardium from E11.5 until birth, with further postnatal maturation (reviewed in [4], [5]. Until the mature conduction system is formed, cardiac action potentials are transduced through the myocardium directly from one cardiomyocyte to another via intercellular gap junctions [6], [7]. In vertebrates, gap junctions are composed primarily of homo- or hetero-hexamers of connexins [8]. The phosphorylation of connexins plays an important role in regulating the different properties of gap junctions, such as the trafficking, assembly/disassembly, degradation and gating of gap junction channels [6], [7]. During mouse embryonic development, five major connexins are expressed in the heart, including Cx45 (the first connexin expressed in developing mouse heart) [9], [10], Cx43 (the main connexin in chamber myocardium) [11], [12] and Cx40 (found in atrial and ventricular chamber myocardium) [13], [14]. Connexin abnormalities contribute to congenital heart defects, illustrating the importance of electrical signal propagation during cardiac development [15].

The *Erbb2* (*Mus musculus v-erb-b2 erythroblastic leukemia viral oncogene homolog 2*) gene encodes a member of the epidermal growth factor receptor (EGFR) family of tyrosine kinases. Due to its extracellular protein structure, the Erbb2 receptor itself does not bind ligands [16]. Instead, it binds other members of EGF receptor family (Egfr, Erbb3 and Erbb4) to form heterodimers, stabilising their binding to ligands and enhancing kinase-mediated activation of downstream signalling pathways (such as MAP and phosphatidylinositol-3 kinase) [17]–[19]. Erbb2 is able to form heterodimers with any of the other three EGF receptors and is believed to be the preferred dimerisation partner [20]. In humans, *ERBB2/HER2* is commonly amplified in breast cancer, leading to receptor over expression and driving the activation of downstream pathways that stimulate malignant cell proliferation [21]. *ERBB2/HER2* amplification is a marker of aggressive disease, although the development of the trastuzumab monoclonal antibody targeting the ERBB2/HER2 receptor has revolutionised breast cancer treatment regimes, leading to improved survival rates [21].

In addition to its pathological role in breast cancer, *Erbb2* is essential for normal embryonic development. Homozygous knock-out mice lacking *Erbb2* are lethal at mid-gestation (embryonic day 10.5) and die due to severe cardiac defects, including an enlarged heart with thin ventricular myocardium, absent trabeculae and decreased atrioventricular cushions, leading to poor circulation and irregular heartbeat [22], [23]. Conditional *Erbb2* mutation in ventricular cardiomyocytes leads to a severe dilated cardiomyopathy associated with the occurrence of sudden death, partially due to an impaired cardiac conduction system [24]. Absence of *Erbb2* also results in neural defects such as degeneration of motor neurons and absence of Schwann cells (Table 1) [22], [25], [26]. It has been shown that *Erbb2* participates in ventricular conduction system development and maturation via its involvement in cardiac trabeculation [23], [27]. However, the role of *Erbb2* in the atrial conduction system development and atrial action potential propagation is unclear.

**Table 1:** Comparison *Erbb2* null [22], [25] and *l11Jus8* phenotypes.

	*Erbb2* null	*l11Jus8 -/-*
**lethality**	E10.5- E11.0	E12.5-13
**general**	degeneration of motor nerves, absence of Schwann cells, impairment of junctional folds at the neuromuscular synapse	no gross morphological internal organ defects
**cardiac**	absent myocardial trabeculae, thin ventricular wall, enlarged heart, poor circulation, irregular heartbeat, lack of cardiac ventricular myocyte differentiation, decreased endocardial cushion size	no ventricular, AV cushion or OFT defects, thickened, folded atrial myocardium which becomes stretched when atria stop contracting, atria inflated with blood, haemorrhage, heart rate normal but abruptly ceases without progressive decline

From an *N*-ethyl-*N*-nitrosourea (ENU) mutagenesis screen [28] we have isolated a mouse line, *l11Jus8*, carrying a hypomorphic missense mutant allele of *Erbb2*. These mice are homozygous lethal by embryonic day (E)12.5-13 and display morphological defects in the developing atria; similar defects have not been reported in *Erbb2* targeted deletion mouse mutants (Table 1). We show that *l11Jus8* homozygous mutants exhibit compromised atrial function due to defects in atrial electrical signal propagation, leading to an atrial-specific conduction block that does not affect ventricular conduction. As relatively few studies have identified specific defects in atrial development, research on the *l11Jus8* mouse model extends our knowledge of the molecular requirements for atrial function. The *l11Jus8* mouse line reveals a novel role for *Erbb2* during atrial conduction system development, demonstrating the importance of atrial function for embryonic survival.

## Materials and Methods

### Mice

The *l11Jus8* mouse strain was generated by random ENU mutagenesis [29]. Mice were maintained in the Biological Service Facility of the University of Manchester, UK, with local ethical approval and according to UK Home Office requirements (Home Office project 40/2912). Mice used for electrical readings were maintained in the Animal Facility of the Institute of Anatomy, Charles University, Prague, Czech Republic and experiments were carried out in accordance with Czech law governing animal care and experimentation. Mice were out-crossed a minimum of 6 times to 129S5/SvEvBrd animals, although the use of the Chr11 balancer ensured that the candidate region on chromosome 11 remained heterozygous for C57BL/6 and 129S5/SvEvBrd DNA in viable animals. The *K14-agouti* transgene integrated on the balancer chromosome [29] allowed the identification of animals without molecular genotyping. Embryo genotyping to distinguish between the 129S5/SvEvBrd and C57BL/6 chromosomes was performed by PCR analysis of genomic DNA using STS markers D11Mit4, D11Mit35, D11Mit327 and D11Mit99. STS marker sequences were obtained from the Ensembl database.

### Next-Generation Sequencing

The Next-Generation Sequencing was performed in the Manchester Centre for Genomic Medicine (Manchester Academic Health Science Centre). Genomic DNA was isolated from a single E12.5 homozygous *l11Jus8* embryo (C57BL/6J background) using ISOLATE II Genomic DNA Kit (Bioline). 1 µg of DNA was used to create the genomic library using the TruSeq DNA Sample Prep Kit. Whole genome sequencing was performed on one lane of a HiSeq 2500 sequencer (Illumina, San Diego, CA, USA), following the manufacturer's protocols. Reads were mapped to the GRCm38/mm10 mouse genome using BWA mapping software [30] and analysed using online Galaxy sequencing analysis tool (main.g2.bx.psu.edu; [31]–[33]). Positions between 69–104 Mb on mouse chromosome 11 were selected and MAQ model with default parameters was applied to identify positions with nucleotides different from the reference. Ensembl Variant Effect Predictor [34] was used to annotate SNPs and eliminate known SNPs. Novel non-coding SNPs were inspected manually to identify those in RNA genes (Table S1). Sequence variants were confirmed in exome sequence data from a single heterozygous *l11Jus8* embryo. Exome sequencing was performed at the University of Liverpool Centre for Genomic Research. Sanger sequencing was performed on an additional 3 homozygous mutant *l11Jus8* embryos using BigDye v3.1 reagents (Applied Biosystems) in the University of Manchester Sequencing Facility according to manufacturer's instructions to verify base changes in coding regions and non-coding RNA genes. Only those variants confirmed by Sanger sequencing in an additional 3 mutant embryos were considered as candidate mutations. Primers for Sanger sequencing: Med31-Forward AGTAGTCCAGCGGTTGGTTG; Med31-Reverse TCATTTTCCATTCCCACACA; Nf1-Forward CCAGCAACTCAATAATGAGCC; Nf1-Reverse TCAAGTTGTGCACCATGCTAG; Erbb2-Forward TCCCTCTGTTCCCTTGTCTG; Erbb2-Reverse AACCCCCAAAGCACATACCT.

### Expression analysis

To test for the expression of *Erbb2* genes in heart tissues, atria, ventricles and erythrocytes were isolated from E12.5 embryos, collected and stored at −20°C in RNAlater solution (Ambion). RNA was isolated from tissue samples (2 hearts were pooled together prior to isolation) using Isolate RNA mini kit (Bioline; genomic DNA degradation step is included in the protocol) and the cDNA was prepared with Tetro cDNA Synthesis kit (Bioline). RT-PCR was performed with following primers: Erbb2-Forward ATGGACAGCACCTTCTACCG; Erbb2-Reverse GGAGCAACGTAGCCATCAGT; Prpf8-Forward TCCTGGACTTATTGGTGAAGTG; Prpf8-Reverse GACACGCCAGCCTTTATCAT. Primers were designed in different exons to allow the transcript to be distinguished from genomic product. Expected products sizes for *Erbb2*: 392 bp for cDNA, 607 bp for genomic DNA; for *Prpf8*: 464 bp for cDNA, 749 bp for genomic DNA. The *Erbb2* fragment was gel purified and sequenced to confirm it was specific for *Erbb2*.

### Protein Structural Modelling

The three dimensional structure of residues 713-1020 of mouse Erbb2 were predicted using homology modelling. The sequences of mouse Erbb2 and human ERBB2 were aligned using ClustalW [35], and structure predicted by Modeller [36] using the known human ERBB2 structure (PDB id 3CRD) [37] as a template. Twenty models were built, and the one with best score was used for further analysis.

**Figure 1:**
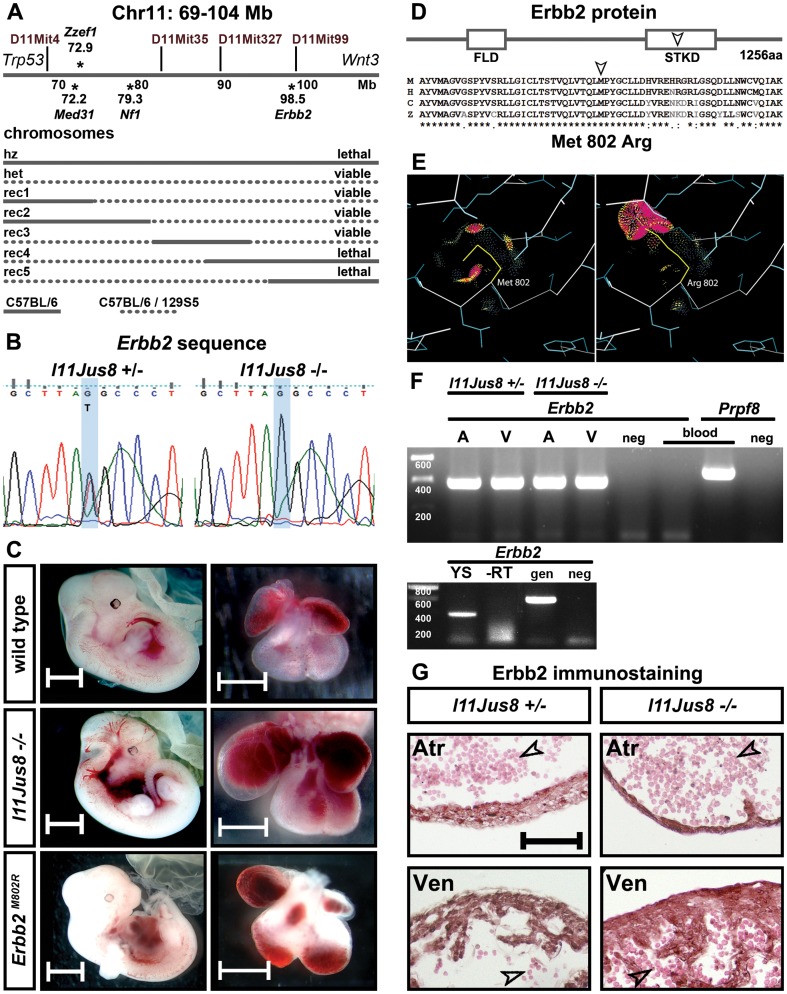
Analysis of DNA, protein, and morphological defects in the *l11Jus8* mutant mouse. (**A**) Three coding mutations found in the 35 Mb candidate intervals on mouse chromosome 11 are located in the *Med31*, *Nf1* and *Erbb2* genes. Only recombinant embryos with the C57BL/6 homozygous DNA fragment inherited from the mutagenised parent which contained the *Erbb2* mutation were lethal at weaning, whereas embryos with homozygous *Med31* and *Nf1* and heterozygous *Erbb2* mutations were viable. Straight and dotted lines depict homo- and heterozygous DNA, respectively. (**B**) Confirmation of the T98,433,986G base change in the *Erbb2* gene in *l11Jus8* mutants by Sanger sequencing. (**C**) Phenotype of wild type, homozygous *l11Jus8* and *Erbb2^M802R^* mutant embryos and hearts. Scale bars: 2 mm for embryos, 500 µm for hearts. (**D**) Erbb2 protein structure: FLD - Furin-Like Domain, STKD - Serine/Threonine-Tyrosine Kinase Domain. Partial sequence of the STK domain (60 amino acids around the mutation point) is shown for mouse (M), human (H), chick (C) and zebrafish (Z) Erbb2 protein. Asterisks indicate conserved amino acids, colon and period indicate conservation between groups of strongly and weakly similar properties, respectively. Arrowhead demarcates the location of the amino acid change. (**E**) Predicted changes in the protein structure of the Erbb2^M802R^ conserved kinase catalytic domain based on the reported crystal structure of the Erbb2 protein. (**F**) Top panel: expression of the *Erbb2* mRNA in atria (A) and ventricles (V) of hetero- and homozygous *l11Jus8,* and embryonic erythrocyte (blood) samples. *Prpf8* mRNA expression is used as a positive control for cDNA isolation in blood sample. Bottom panel: *Erbb2* expression in the yolk sack (YS) of homozygous *l11Jus8* embryo, “-RT” control, genomic DNA (gen) control. A no template negative control (neg) is shown in both panels. (**G**) Erbb2 immunohistochemistry in heterozygote and *l11Jus8* atria (Atr) and ventricles (Ven). Embryonic erythrocytes within the cardiac chambers are indicated with an arrowhead. Scale bar  =  200 µm.

In order to predict the likely effects of the Met-802-Arg point mutation, hydrogen atoms were added using Reduce software [38]. The mutation was modelled using KiNG software [39]. All low energy side chain conformation (rotamers) [40] were modelled, and their interactions with the rest of the protein structure assessed using the all-atom contact method [41]. Substantial van der Waals overlaps were found with all rotamers, indicating that the mutant protein cannot fold to the same native structure without additional structural accommodation. The rotamer with the smallest volume of van der Waals overlaps is shown in Figure 1.

### Histology

Tissues were fixed in Bouin's fixative in PBS and dehydrated through an ethanol gradient. Samples were embedded in paraffin (Paraplast, Sigma), sectioned at 7 µm thickness and stained with Harris' hematoxylin and 1% eosin Y.

### Immunostaining

Paraffin-embedded tissue sections were de-waxed and rehydrated prior to immunohistochemistry. Antibodies detecting α-Smooth Muscle Actin (1∶450, Sigma-Aldrich), Laminin α1 (1∶1000, gift from Prof Ray Boot-Handford, University of Manchester, UK), Fibronectin (1∶50, Santa Cruz Biotechnology, Heidelberg, Germany), Phospho-histone H3 (1∶1000, Millipore, Watford, UK), Erbb2 (1∶100, gift from Prof Keith Brennan, University of Manchester UK), and Activated Caspase-3 (1∶500, R & D Systems, Abingdon, UK) were incubated for a minimum of 1 hour at room temperature. Samples were then washed in PBS, incubated with secondary antibody (biotinylated horse anti-mouse 1∶250, biotinylated goat anti-rabbit 1∶1000, biotinylated horse anti-goat 1∶200), and protein localisation visualised using DAB Peroxidase Substrate Kit (SK-4100, Vector Labs). Immunostaining detecting Connexins 40 and 43 was done using Cx40 (1∶100, Santa Cruz) and Cx43 (1∶100, Sigma) antibodies on PFA fixed, paraffin-embedded sections. Antigen retrieval was by boiling in sodium citrate buffer (10 mM pH 6.0) for 20 minutes, samples were then blocked with 3% goat serum for 1 hour at room temperature before incubating with the primary antibody O/N at 4°C (Cx43 1∶100 in PBS and Cx40 1∶100 in 10% goat serum/PBS). Samples were washed the next day in PBS, and incubated with secondary antibody (Cx43 - goat anti-rabbit-FITC 1∶250, Sigma 2 hours at room temperature; Cx40 - anti-goat biotinylated, 1∶250, Vector Labs for 2 hours at room temperature, followed by a 30 minute incubation with Streptavidin-Cy3, 1∶500, GE Healthcare).

For visualisation of cell nuclei, sections were mounted with Vectashield mounting medium containing DAPI (Vector Labs) immediately followed by imaging.

For detection of necrotic cells, freshly dissected hearts were placed in 1 µg/ml Propidium Iodide solution in PBS and incubated for 30 minutes at room temperature, washed for 5 minutes in fresh PBS and imaged using fluorescent microscopy.

### *In situ* hybridization

Whole mount *in situ* hybridization was carried out as previously described [42]. The *Myl4* probe was generated by cloning a PCR product containing the *Myl4* cDNA into the PCRII dual promoter plasmid (Invitrogen). PCR primer sequences were *Myl4* exon2: GCAACCGACAGTGTCCATATA; *Myl4* 3′UTR: CATGTGAGTCCAATACTCCGT.

### Optical mapping

Embryos were decapitated; hearts and the adjacent posterior thoracic wall were isolated and stained with voltage-sensitive dye 4-{-[2(di-n-butylamino)-6-napthyl]vinyl} pyridiniumin (di-4-ANEPPS, Invitrogen) in Tyrodes solution for 5 minutes at room temperature [43]. E12.5 embryos with visible thoracic haemorrhage and/or yolk sac vascular defects (6/21) were excluded from analysis. Optical mapping was performed at 37°C in oxygenated Tyrodes with added blebbistatin (1∶1000 of 14 mM stock in DMSO) to stop muscle contractions and heart movements. Recordings were done with Ultima L high-speed camera (SciMedia Ltd., Japan) at 0.25, 0.5 and 1 kHz and the analysis of recordings resulting in the generation of spatio-temporal epicardial activation maps was performed using the BV_Analyzer software. The protocol and processing algorithms for generation of activation maps were described in detail previously [44]. At least three recordings were made for each sample. Activation maps were compared together with the rhythm strip of atrial and ventricular rate between genotypes essentially as described [45]. Parameters analysed presence, direction and extent of atrial and ventricular activation and propagation. Gaps in atrial signal propagation were detected by varying threshold of signal/noise ratio during electrical map reconstruction (Figure S1, S2). The presence of the gaps was judged by two parameters: first, a map was considered to contain “gaps” if there was an area without clear signal found at the threshold level when significant noise was still observed in the rest of the image (Figure S1). Secondly, gaps were confirmed by the absence of the signal in one area of the atrium and presence in the other one (Figure S2B–C and F–G).

## Results

### *l11Jus8* mouse line: mutation isolation and analysis

The *l11Jus8* mutant mouse line was created by random ENU mutagenesis and isolated from a balancer chromosome screen for mutants with embryonic lethality [28], [29]. The *l11Jus8* line was selected for further study because it displayed embryonic lethality at E12.5 arising from suspected cardiac defects, including vascular and cardiac haemorrhage. Due to the use of a balancer chromosome in the mutagenesis screen, the candidate interval for the *l11Jus8* mutation was known to be within a 35 Mb region of mouse chromosome 11 between the *Trp53* and *Wnt3* loci [29]. Since homozygous mutant *l11Jus8* embryos do not survive past day E13.5 of embryonic development, we hypothesised that an important protein function would be disrupted in these mutants. In order to find the causative mutation, we isolated genomic DNA from homozygous *l11Jus8* embryos, performed genomic Next Generation Sequencing (NGS), and used bioinformatic analysis to identify single nucleotide variations within the 35 Mb candidate interval. Using the GRCm38 mouse genome assembly, three coding changes on mouse chromosome 11 were found: T72,213,810C in the *Med31* gene resulting in Tyr-57-Cys missense change, T79,447,501A located within the *Nf1* gene resulting in a Met-1113-Lys missense mutation and T98,433,986G within the *Erbb2* gene causing a Met-802-Arg missense change (Figure 1A, Table S1). These changes were confirmed by Sanger sequencing on three additional homozygous *l11Jus8* mutant embryos. Additionally, annotated novel and non-coding RNAs (microRNAs, lncRNAs, anti-sense RNAs) were also checked for sequence variations specific to the *l11Jus8* mutant. Predicted base changes within RNA genes were not confirmed by further Sanger sequencing on additional mutant embryos (Table S1).

To determine which sequence variant best represented the *l11Jus8* causative mutation, we bred animals carrying recombinant chromosomes to *l11Jus8* heterozygotes, and analysed the viability at weaning of offspring carrying homozygous C57BL/6 DNA (inherited from the mutagenised parent) in smaller intervals within the candidate region. Based on the analysis of genomic DNA from recombinant animals, only the *Erbb2* mutation is a valid genetic candidate for causing the *l11Jus8* phenotype (Figure 1A), because lethality was only seen in recombinants homozygous for the *Erbb2* mutation. We have confirmed that no animals homozygous for the *Erbb2* mutation survived to weaning age, showing a significant deviation from expected Mendelian frequency (Chi-squared test; Table S2). Sanger sequencing demonstrated that the *Erbb2* mutation was present in homozygous *l11Jus8* mutants (Figure 1B). Dissections at E12.5 confirmed that embryos homozygous for the mutation in the *Erbb2* gene exhibit the *l11Jus8* mutant phenotype (Figure 1C) while heterozygous animals were viable, fertile, and showed no defects during development. Animals double homozygous for both the *Med31* and *Nf1* missense mutations were viable at weaning age (3 pups in 1 litter). Additionally, a non-coding mutation in *Zzef1* previously identified in this mouse line [46] was excluded as a candidate based on recombination mapping.

The mutation in the *Erbb2* gene is located in the conserved Serine-Threonine/Tyrosine protein kinase catalytic domain (Figure 1D). Utilising the reported crystal structure of the human ERBB2 protein (PDB id 3RCD [37]), we predict from an analysis of side-chain rotamer conformations [40] that the M802R amino acid change will create van der Waals clashes, impeding folding and likely causing a loss of kinase catalytic function (Figure 1E). It is not known how the M802R affects Erbb2 protein function. We did not detect any differences in the phosphorylation of Erk, one Erbb2 target, in *l11Jus8* mutants (Figure S3), so at present cannot determine the biochemical nature of the M802R mutation.

To confirm that *Erbb2* is expressed in cardiac tissues and to determine if the missense mutation affects gene expression, we performed RT-PCR with cDNA prepared from RNA isolated from E12.5 atria and ventricles of *l11Jus8* heterozygote and homozygote hearts. Since the heart lumen is filled with erythrocytes that are difficult to separate completely from the surrounding cardiac tissue, as a control we also prepared cDNA from embryonic erythrocytes collected during dissections. Clear bands indicating the expression of *Erbb2* were found in atrial and ventricular samples of both genotypes but not in the erythrocyte sample (Figure 1F top panel). The expression of the *Prpf8* gene (encoding a core component of the spliceosome) [47] was used as a positive control to confirm the presence of cDNA in erythrocyte sample. We also identified *Erbb2* expression in the developing E12.5 wild type yolk sac (Figure 1F bottom panel). To determine if Erbb2 protein distribution was altered in *l11Jus8* mutants, we examined the localisation of Erbb2 in E12.5 heterozygote and homozygous mutant hearts. No alterations in the expression pattern in the ventricles or atria were detected (Figure 1G). Notably, Erbb2 was expressed throughout the cardiac tissue, but not within blood cells, supporting our RT-PCR data. The presence of Erbb2 protein in *l11Jus8* mutants supports our hypothesis that the M802R mutation does not create a null allele.

### Morphological characterisation of the *l11Jus8* mutant phenotype

**Figure 2:**
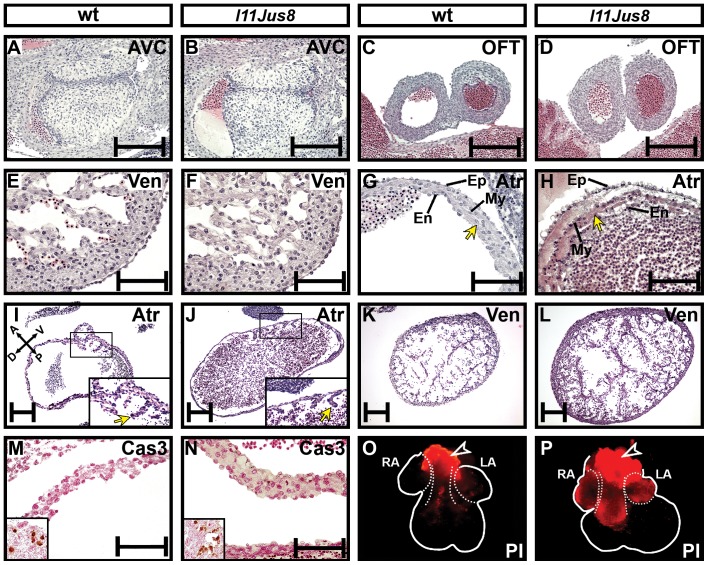
Effect of the homozygous *Erbb2^M802R^* mutation on heart morphology in *l11Jus8* hearts. (**A**–**L**) Representative H and E stained coronal sections of E12.5 hearts. (**A**–**B**) Atrioventricular cushions, (**C**–**D**) dorsal outflow tract, (**E**–**F**) ventricles, (**G**–**H**) atria. Arrows in (**G**–**H**) point to the atrial wall. AVC, atrioventricular cushion; OFT, outflow tract; Ven, ventricle; Atr, atrium; Ep, epicardium; En, endocardium; My, myocardium. (**I**–**J**) Longitudial atrial and (**K**–**L**) ventricular sections; homozygous *l11Jus8* heart had distended atria. Embryos in **I**–**L** are littermates. Magnified areas in (**I**–**J**) show the developing pectinate muscles (arrows). (**M**–**N**) Activated Caspase 3 (Cas3) staining of atrial wall at E11.5. Inset shows positive activated Caspase3 staining from trigeminal ganglion on same embryo section. (**O**–**P**) Propidium Iodide (PI) labelling of the necrotic cells in E12.5 hearts. RA, right atrium; LA, left atrium. Arrowheads point to the unspecific PI staining resulting from cells of the OFT damaged during dissection. Scale bars: 200 µm in **A**–**H** and **M**–**N**, 50 µm in **I**–**L**.

Heterozygous *l11Jus8* embryos were viable and had no overt developmental defects (Figure S4A, D, B, E). Homozygous mutants were lethal with embryonic death observed between days E12.5-13 of development; mutants exhibited severe haemorrhages in the thoracic cavity and head (Figure 1C). Mutant yolk sacks (YS) display reduced YS vessel volume becoming progressively more severe from E11.5 – E12.5, resulting in visible absence of blood in YS vessels (Figure S5). Notably, yolk sack defects were only visible in the embryos with haemorrhages in the thoracic cavity. Approximately 29% of mutant embryos possessed yolk sac defects at E12.5, indicating that this aspect of the phenotype is not fully penetrant, while lethality by E13.5 was fully penetrant. Histological examination of *l11Jus8* mutant embryos at E11.5 revealed subtle defects in atrial wall structure; no other cardiac abnormalities were detected at this stage, and hearts were beating visibly during dissections (data not shown). Because mice homozygous for a targeted deletion of *Erbb2* display a reduction in endocardial cushion size [22], we analysed atrioventricular cushion development in *l11Jus8* mutant embryos at E12.5 (Figure 2A–B) but found no differences as compared to control littermates. Similarly, no defects were observed in outflow tract (OFT) vessel septation (Figure 2C–D), despite the reported role for *Erbb2* in this process [48]. *Erbb2* knockout embryos also display a lack of ventricular trabeculation leading to death by E11.0 [22], however *l11Jus8* mutant embryos survive past this time point, and have clear evidence of trabeculation at E12.5 that is indistinguishable from control littermates (Figure 2E–F).

Although ventricular myocardium, outflow tract and atrioventricular cushion morphology was normal, 5/15 homozygous *l11Jus8* E11.5 embryos exhibited collapsing of atria such that the chamber lumen appeared decreased in size and the atrial myocardium appeared thickened and irregularly folded (data not shown). Highly eosinophilic stained patches containing fragmented irregularly shaped cell nuclei were present, and the epi- and endocardium were detached from the myocardium; by E12.5 these changes were more pronounced (Figure 2G–H arrows). In mutant hearts with distended atria (Figure 1C), the atrial wall was noticeably thinner in comparison to wild type (Figure 2I–J) while ventricles remained unchanged (Figure 2K–L). The developing pectinate muscles, found specifically in the atria, were present in both heterozygous and homozygous mutant atria (magnified areas in Figure 2I–J). Aside from cardiac abnormalities, no defects in the development of internal organs or embryonic vasculature were found in *l11Jus8* mutants (Figure S4C, F). Notably, the atrial phenotype observed in homozygous *l11Jus8* mutants cannot be examined in *Erbb2* null embryos (phenotype summarised in Table 1) [22], [25] due to the early gestation lethality of *Erbb2* null mutants.

### Molecular characterisation of the *l11Jus8* mutants

To further characterise the *l11Jus8* mutant phenotype, we analysed the expression of *Myl4*, a marker of cardiomyocyte differentiation, in wild type, *l11Jus8* heterozygous and homozygous E10.5 embryos (Figure S6A–C). We also performed immunohistochemistry on atrial cross-sections from wild type, *l11Jus8* heterozygous and homozygous E12.5 hearts for Smooth Muscle α-Actin (smooth muscle cell marker), Laminin α 1 (basement membrane component), Fibronectin (cardiac ECM component) and E11.5 hearts for phospho-histone H3 (marker of cell proliferation). Surprisingly, expression of all markers was similar between all three genotypes suggesting the presence of correct cell types and cellular functions in *l11Jus8* homozygous mutant hearts (Figure S6D–O).

DAPI staining of cell nuclei in E12.5 atrial sections (Figure S6P–R), revealed signs of fragmented DNA in the eosinophilic patches in *l11Jus8* mutants (shown in Figure 2H), which may indicate extensive cell death in these areas. We tested this hypothesis and found that activated Caspase3 expression was identical between wild type and *l11Jus8* mutants (Figure 2M–N), suggesting that there was no increased apoptosis in *l11Jus8* mutants. However, staining with propidium iodide revealed necrotic cells in E12.5 *l11Jus8* mutant atria (3/6 mutant hearts) but no necrosis in heterozygous atria (0/10 heterozygous hearts; Figure 2O–P).

### Cardiac function in *l11Jus8* mutant embryos

Since the *l11Jus8* hearts do not show significant defects at the morphological or molecular level but the embryos display mid-gestation lethality consistent with cardiac defects, we next investigated heart function in the mutant embryos. The heart rate of E10.5, 11.5 and 12.5 embryos was assessed visually during dissections and on ultrasound movies of embryos *in utero.* No differences were observed between wild type, heterozygous and viable homozygous *l11Jus8* embryos (data not shown). However, only 8/10 E11.5 and 4/12 E12.5 homozygous *l11Jus8* embryos were viable with a heartbeat present at the time of dissection (Table S3). Moreover, during dissections *l11Jus8* mutant hearts with normal macroscopic atrial morphology always contracted normally but hearts with distended atria (confirmed via genotyping as homozygous *l11Jus8* mutants) only had ventricles contracting; atria in these hearts either did not contract at all or in rare cases, twitched slightly but lacked proper blood movement through the heart.

### Optical mapping of action potential propagation

**Figure 3:**
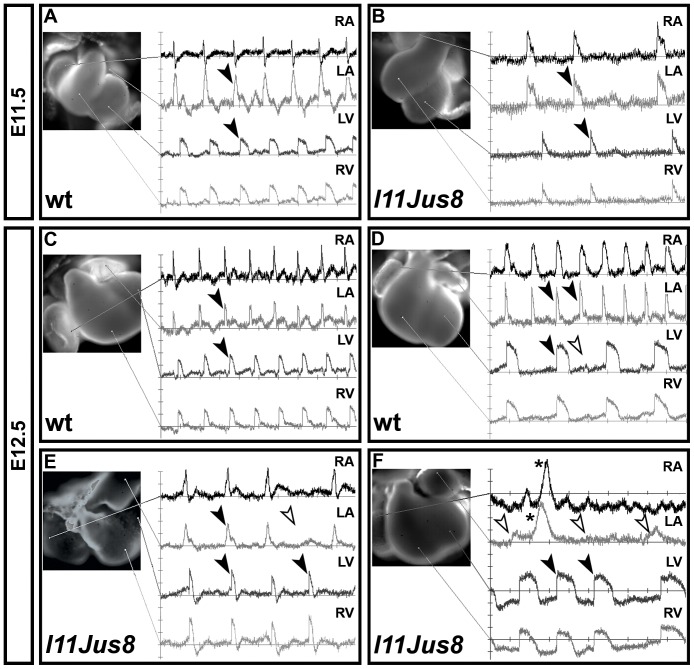
Optical mapping of the cardiac electrical activity obtained with di-4-ANEPPS voltage sensitive dye. (**A**–**B**) and (**C**–**F**) show reading of E11.5 and E12.5 hearts, respectively. The location from which the electrical reading was taken is shown with lines onto the cardiac dissection image. Black arrowheads point to matched atrial and ventricular signals. (**D**) White arrowhead indicates absent signal in left ventricle corresponding to a present signal in left atria (black arrowhead), indicative of AV block. (**E**–**F**) White arrowheads indicate absent atrial signals corresponding to present ventricular signals (A block), a phenotype only present in mutant samples. Asterisks on RA and LA traces in (**F**) indicate signal described in **Figure 5C**. RA, right atrium; LA, left atrium; LV, left ventricle; RV, right ventricle.

To investigate cardiac function of the *l11Jus8* mutants in detail, we performed optical mapping of the electrical activity in wild type and mutant hearts using a voltage-sensitive dye di-4-ANEPPS to visualise the propagation of the electrical signal through the tissue [43]. In E11.5 hearts of all three genotypes the electrical signal was detected in both atria and ventricles (Table 2; Figure 3A–B; signal in heterozygous hearts not shown). Occasionally, partial atrioventricular block (AV block, defined as signal present in atria but not observed in ventricles) was detected (AVb in Table 2). AV block is often found in optical mapping experiments and results from experimental manipulations [43]. Notably, the *l11Jus8* mutant hearts did not display distended atria at E11.5 (Table 2). In E12.5 samples, 9/21 (43%) homozygous *l11Jus8* hearts had distended atria and did not contract; mapping these hearts did not result in clear reading of the electrical signal and they were excluded from further analysis (labelled “distended” in Table 2). Only *l11Jus8* mutant E12.5 hearts without atrial distension were used in subsequent analysis of electrical activity. At this stage wild type hearts displayed consistent electrical activity in both the atria and ventricles (Figure 3C), or the presence of AV block (Figure 3D white arrowhead). Overall, AV block was found in 58%, 46% and 50% of wild type, heterozygous and homozygous E12.5 *l11Jus8* hearts, respectively (AVb in Table 2). Surprisingly, we also found partial (Figure 3E white arrowhead) or full atrial block (Figure 3F white arrowheads): while the electrical signal was present in ventricular myocardium (Figure 3E–F black arrowheads), the corresponding atrial signal was not detected. Atrial block was observed in 2/50 (4%) heterozygous and 6/12 (50%) homozygous *l11Jus8* hearts (Ab in Table 2) but not in any of the 29 wild type hearts analysed. An atrial block phenotype has not been described before in wild type mouse or chick hearts, or *Cx40* mutant mouse hearts (55 chick hearts ages 3–10 days; 19 wild type mouse hearts, 12 *Cx40*+/− and 18 *Cx40*−/− hearts; [45], [49]). Notably, the percentage of absent atrial peaks (as compared to the presence of ventricular peaks) was 13% and 36% in two heterozygous embryos. However, in *l11Jus8* homozygous hearts a range of 3.3–100% of atrial peaks were missing (one sample had full atrial block with no atrial signals detected while regular ventricular signal was observed). Statistical analysis shows that the proportion of embryos with atrial block is significantly different between all genotypes in pair-wise comparisons (heterozygous and homozygous *l11Jus8* samples Chi square, p<0.0001). The observation that atrial block was present in homozygous *l11Jus8* mutant hearts lacking yolk sac vessel defects, cardiac distension, or haemorrhage suggests that atrial electrical alterations precede or occur independently of morphological abnormalities of the atria, yolk sack or necrosis in the mutant hearts. Thus impaired atrial electrical function is the most likely reason for embryonic lethality in *l11Jus8* mutants.

**Table 2:** Numbers of distended hearts, hearts with atrioventricular block (AVb), atrial block (Ab), and atrial signal conduction gaps in optical mapping experiments.

	E11.5			E12.5		
	Wild type	het	*l11Jus8*	Wild type	het	*l11Jus8*
**embryos (total)**	6	6	6	29	50	21
**distended heart**	0	0	0	0	0	9
**AVb**	2	1	1	17	23	6 (of 12)
**Ab**	0	0	0	0	2	6 (of 12)
**A gaps**	0	0	3	0	7	10 (of 12)

**Figure 4:**
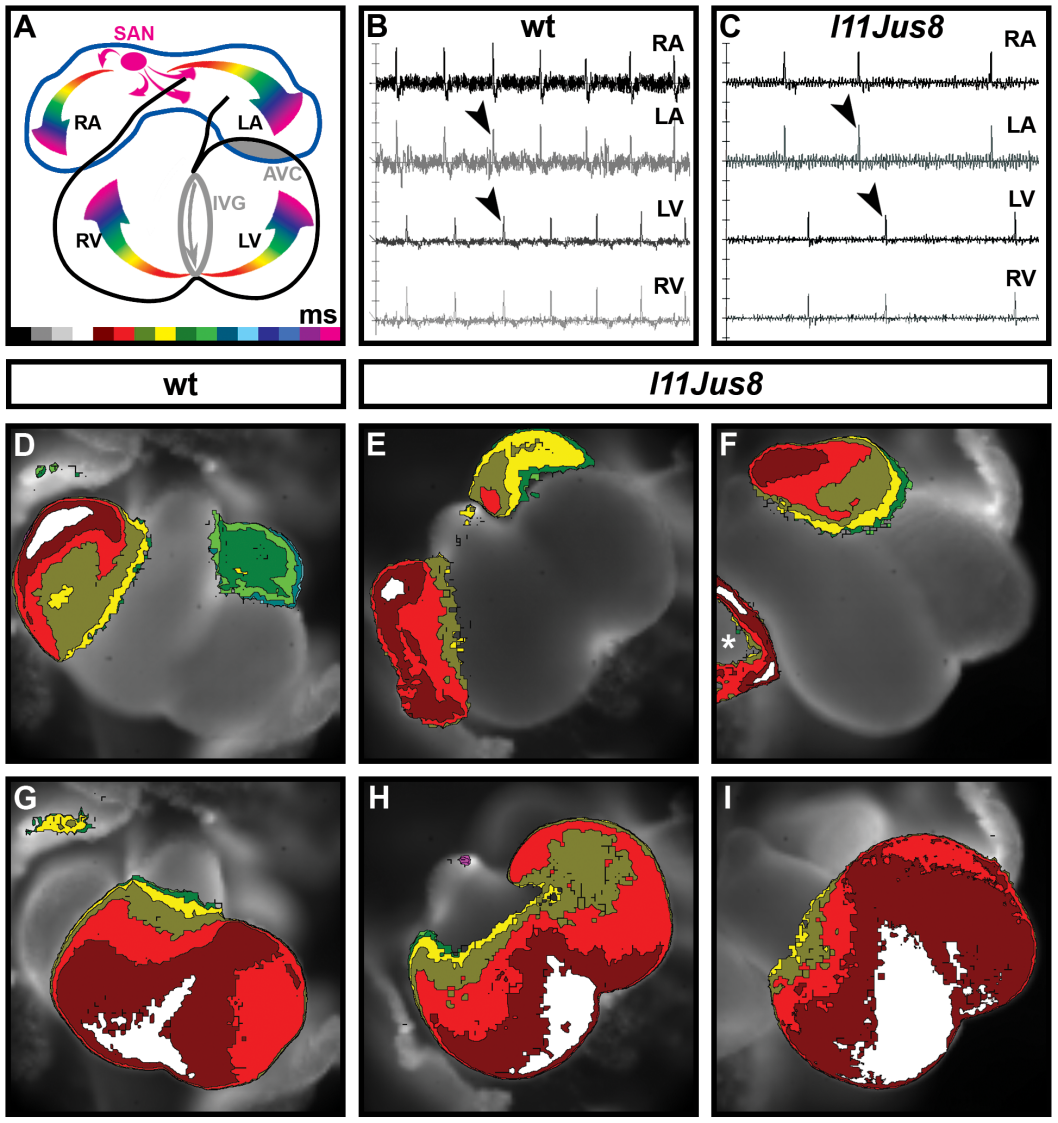
Mapping of electrical signal propagation in E11.5 hearts. (**A**) Pattern of wild type electrical signal propagation in the E11.5-12.5 heart. Different colours encode the time scale of the signal conduction; each colour represents a one-millisecond interval with black as the initial time point and fuchsia as the final time point. (**B**–**C**) Derivatives of the signal recorded in wt and homozygous mutant *l11Jus8* E11.5 hearts. Black arrowheads point to the peaks used for map generation. (**D**–**F**) Optical maps showing spatio-temporal propagation of the electrical signal through the atria. Asterisk on (**F**) points to the gap in normal electrical activity in homozygous mutant *l11Jus8* atria. (**G**–**I**) Optical maps showing electrical signal propagation through the ventricles. Maps are similar for all genotypes. SAN, sinoatrial node; RA, right atrium; LA, left atrium; LV, left ventricle; RV, right ventricle; AVC, atrioventricular canal; IVG, interventricular groove.

We next investigated the spatiotemporal propagation of the electrical signal through the atrial and ventricular tissue using maps reconstructed from the optical recordings [43]. Electrical signals travel through the developing heart in a characteristic pattern (Figure 4A [45], [50], and alterations in the signal propagation pattern could indicate cardiac dysfunction. Signal propagation maps were reconstructed using the derivatives (Figure 4B–C) calculated from the graphs of the electrical activity shown previously (Figure 3A–B). At E11.5 in hearts of all genotypes without atrial block, the electrical signal originated in the roof of the right atrium, spread within 3–5 ms through the right atrium, and travelled with a slight delay (approximately 2 ms) across the left atrium (Figure 4D–F). After 50–130 ms delay due to passing through the slow-conducting AV canal tissue (AVC), the signal appeared in the interventriclular groove (IVG) from where it moved laterally along ventricles (Figure 4G–I). In E11.5 wild type, heterozygous and 3/6 homozygous *l11Jus8* hearts complete atrial activation maps without gaps were reconstructed (Figure 4D–E). However, in 3/6 homozygous *l11Jus8* E11.5 hearts, gaps indicating regions of tissue lacking electrical conduction (further defined in Materials and Methods and Figures S1 and S2) were found (asterisk in Figure 4F), implying aberrant signal propagation in mutants. Ventricular signal propagation was complete without gaps in all samples (Figure 4G–I).

**Figure 5:**
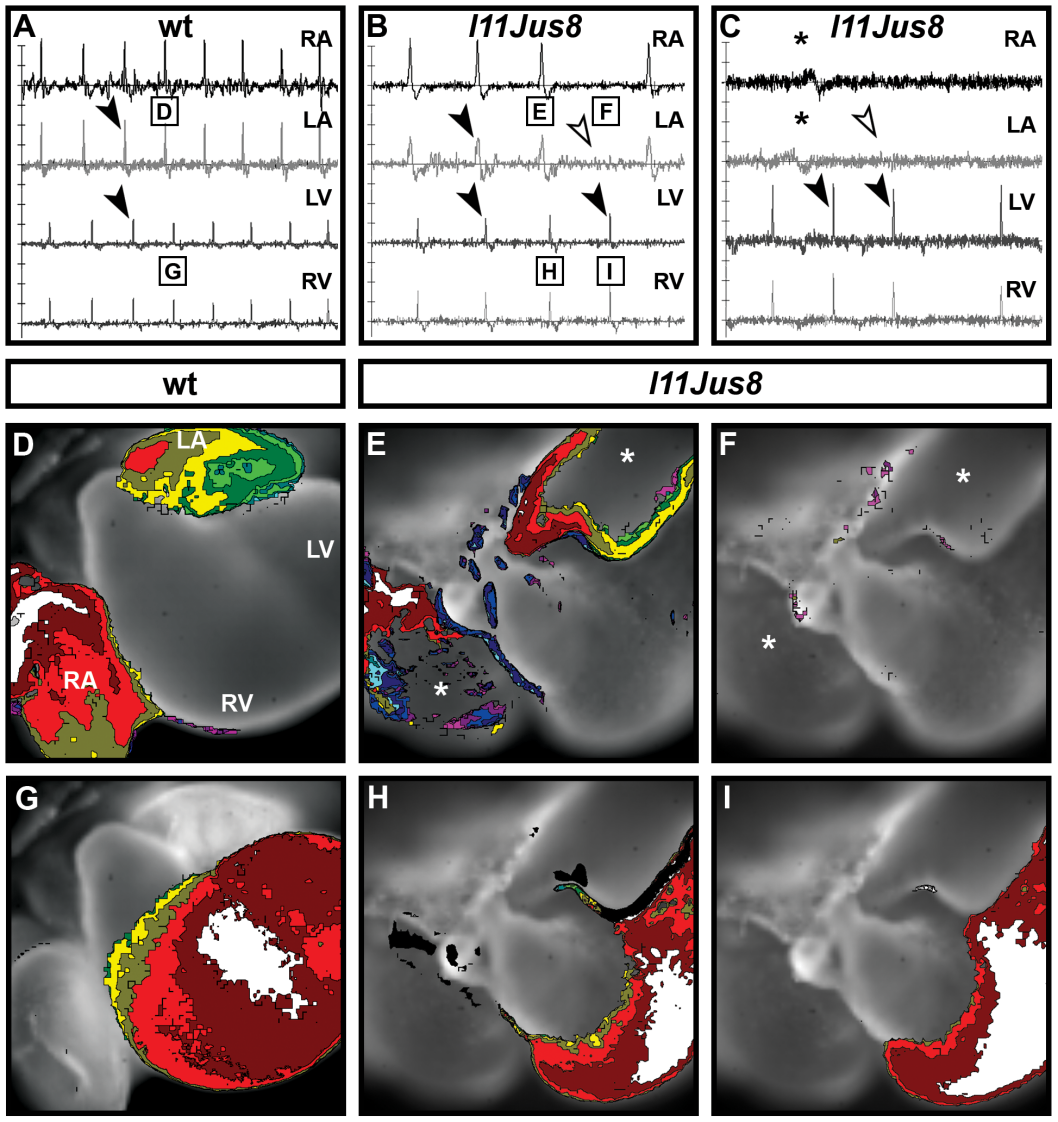
Mapping of electrical signal propagation in E12.5 hearts. (**A**–**C**) Derivatives of the signal recorded in wt and homozygous *l11Jus8* E12.5 hearts. Black arrowheads point to the present peaks used for map generation, white arrowheads point to the missing peaks. Asterisk on (**C**) depicts the missing derivative for signal present on **Figure 3F**. The derivatives used in each map image are indicated on the electrical traces. (**D**–**F**) Optical maps showing spatio-temporal propagation of the electrical signal through the atria. Asterisks indicate gaps in normal electrical activity. White arrow shows the direction of the signal propagation in wild type and homozygous *l11Jus8* atria. (**G**–**I**) Optical maps showing electrical signal propagation through the ventricles. Maps are similar for all genotypes. Each colour (**D**–**I**) represents a one-millisecond interval with black as the initial time point and fuchsia as the final time point. RA, right atrium; LA, left atrium; LV, left ventricle; RV, right ventricle.

Derivatives of the electrical signal were also used to reconstruct signal propagation maps at E12.5 (Figure 5A–C, corresponding to electrical signal shown in Figure 3C, E, F). In E12.5 hearts, no atrial gaps could be detected in wild type samples (Figure 5D). However, regions of atrial tissue lacking electrical signal were observed in 7/50 (14%) heterozygous (data not shown) and 10/12 (83%) homozygous hearts (Figure 5E–F). The proportion of samples demonstrating atrial conduction gaps is statistically significant when compared between wild type and homozygous *l11Jus8* samples, as well as between heterozygous and homozygous *l11Jus8* samples (p<0.0001). All 6 homozygous *l11Jus8* hearts with partial atrial block exhibited gaps in the maps reconstructed from the detected atrial signals, with the majority of atrial tissue completely lacking electrical activity (Figure 5F). Moreover, all maps reconstructed from the sequence of signals in the same sample showed gaps in conduction (Figure S7). In samples of all genotypes, ventricular propagation of the signal was complete over the entire tissue without gaps in conduction (Figure 5G–I).

During embryonic development, prior to the formation of the mature cardiac conduction system, cardiac action potentials are transduced between cardiomyocytes via intercellular gap junctions [6], [7]. Connexin protein localisation serves as an indicator of cellular gap junctions, and alterations in connexin protein distribution could result in aberrant gap junctions leading to errors in electrical signal propagation in the embryonic heart [15]. Due to the electrical signal propagation defects detected in homozygous *l11Jus8* hearts, we therefore examined the distribution of Connexin 40 and Connexin 43 in wild type and *l11Jus8* mutant hearts. However, in both the atria and ventricles of wild type and *l11Jus8* mutant hearts no differences in the distribution of either protein could be identified (Figure S8). It has been reported that heterozygosity for either Cx40 or Cx43 reduces atrial conduction [51]. Because we detect both Cx40 and Cx43 in mutant hearts, we hypothesise that a loss of Cx40 or Cx43 is not the underlying mechanism for the atrial conduction defects of the *l11Jus8* mutant.

Overall, the optical mapping data above demonstrate that atria of *l11Jus8* mutant hearts begin to lose the ability to conduct electrical signal at E11.5, leading to a block in atrial conduction by E12.5-13. The loss of gap junctions due to abnormalities in connexin protein distribution is unlikely to underlie these conduction defects. We propose that the embryonic lethality detected in *l11Jus8* mutants arises due to atrial conduction failure and subsequent atrial necrosis, as no other significant cardiovascular abnormalities have been found in mutant embryos.

## Discussion

We present data to demonstrate that a missense mutation in *Erbb2* results in a specific defect in atrial electrical conduction during embryonic development. The M802R mutation affects a residue within the protein kinase domain of Erbb2 that is conserved across multiple species, including human. Our analysis of human sequence variations from the 1000 genomes project [52] reveals that although this particular mutation is not found in the dataset, variants at positions nearby are likely to be deleterious. The 1000 genomes dataset contains 68 missense variants within the kinase catalytic domain of the ERBB2 protein (ENSP00000269571), of these 45 are predicted by SIFT [53] to be deleterious, and 46 are predicted by PolyPhen [54] to be probably damaging. Additionally, our structural modelling of the M802R mutation indicates that protein stability may be altered due to side chain conformation clashes.

Although the extracellular domain structure of Erbb2 precludes ligand binding [16], the Erbb2 protein functions through heterodimerisation with other members of the EGFR family to achieve ligand-dependent activation of tyrosine kinase activity [16], [55], [56]. There are a series of five autophosphorylation sites within the Erbb2 cytoplasmic tail, one of which has been demonstrated to negatively regulate Erbb2 signalling [57]. Knock-in mice have been generated carrying mutations within these autophosphorylation sites. Notably, homozygous knock-in animals for Y1028F, Y1144F, and Y1227F Erbb2 missense mutations were viable, with an apparently normal phenotype. Animals carrying a hypomorphic Erbb2 allele expressing 10% of wild-type protein levels *in trans* to a null allele demonstrated perinatal lethality due to impaired diaphragm innervation causing respiratory failure [58]. The Y1028F allele when placed in trans to a null allele was found to rescue the perinatal lethality by reducing Erbb2 protein turnover [58]. Cardiac embryonic development can be assumed to be sufficient in the *Erbb2* hypomorphic mutants due to their survival into the perinatal period. Therefore, the atrial electrical alterations and death detected in the *l11Jus8* homozygous mutant embryos may indicate that the M802R mutation within the kinase domain of Erbb2 severely compromises Erbb2 function in a manner distinct from the autophosphorylation site missense mutations. In comparison to the less severe phenotypes generated from autophosphorylation site mutants, our data indicate that kinase activity is of greater importance than autophosphorylation for Erbb2 function. Many of the *Erbb2* null mutants were analysed on a mixed 129 and C57BL/6 background [22], [25], [26]. The *l11Jus8* mice were also maintained on a mixed 129 and C57BL/6 background, although there are likely to be differences to the prior knockouts in the percentages and areas of the genome that are comprised from each strain. Thus, we cannot formally exclude genetic background differences as a confounding factor in comparing the M802R allele to other *Erbb2* mutant alleles.

Because the Erbb2 receptor lacks ligand-binding activity, its signalling function must be mediated through interactions with other family member proteins. Therefore, the phenotype we detect in the *l11Jus8* mutant mouse might arise due to impaired signal transduction specific to one interacting partner. An examination of the null phenotypes of *Egfr/Erbb1*, *Erbb3* and *Erbb4* mutant mice indicates that the cardiac defects present are dissimilar to the *l11Jus8* mutant [56]. For example, *Erbb4* null mutants display cardiac trabeculation defects and lethality at E10.5 [59] that is similar to the *Erbb2* null phenotype. *Erbb3* null mutants display a failure to form endocardial cushions, linked to lethality at E13.5 [25]. *Egfr/Erbb1* null mutants display early embryonic lethality, but this is not primarily linked to cardiac dysfunction [60], [61]. Thus, the null phenotypes of other EGFR family proteins are not identical to the *l11Jus8* phenotype, although genetic background may also contribute to phenotype differences. A more detailed investigation of the function and downstream signalling activities of the other EGFR family members in *l11Jus8* mutants may determine if moderation of the function of a specific family member or downstream signalling effector contributes to the *l11Jus8* phenotype.

It is unclear why the electrical conduction defect seen in the *l11Jus8* mutants is restricted to the atria, as we have shown that *Erbb2* is expressed in both the atria and ventricles of the developing heart. Prior studies on a zebrafish *Erbb2* mutant demonstrated an aberrant pattern of electrical activity which indicated the ventricular conduction system failed to develop [27]. Additionally, the EGFR ligand Neuregulin 1 (Nrg1) has been shown to promote cardiomyocyte differentiation into cardiac conduction system cells [62]. Hearts cultured with exogenous Nrg1 demonstrate an altered pattern of ventricular conduction activity, however atrial electrical mapping was not presented in the study [62]. Nrg1 treatment of adult rat ventricular cardiomyocytes in primary culture increases focal adhesion formation, resulting in synchronous beating [63], demonstrating another link between *Erbb2* and ventricular conduction. It is noted that *Nrg1* has higher expression in the ventricles and *Nrg2* expression levels are greater in the atria [62]. However, a critical role for *Nrg2* in atrial conduction system development can be ruled out from studies on the *Nrg2* null mouse, which is viable and presents no cardiac conduction system defects [64].

Embryonic cardiac defects, including atrial conduction failure, are not found in mice with cardiomyocyte-specific deletions of *Erbb2* [24], [65]. At present our experiments cannot determine whether a non-cardiomyocyte tissue source requires Erbb2 function for cardiac conduction system development, or whether the M802R missense mutation interferes with a key factor for cardiac conduction system function in a manner that is not replicated in null models. In addition to the embryonic requirement for Erbb2 for cardiac function, it has been proposed that Erbb2 functions in a cardioprotective capacity in adult cardiomyocytes [66]. Notably, the use of the anti-cancer drug trastuzumab, an ERBB2 specific-inhibitor, causes cardiotoxicity and congestive heart failure in some breast cancer patients [55]. Similarly, the deletion of *Erbb2* in adult cardiomyocytes causes early cardiac dysfunction and severe dilated cardiomyopathy in the absence of readily demonstrable cardiomyocyte apoptosis [24], [65]. In humans and mice dilated cardiomyopathy contributes to impairment of the conduction system, causing arrhythmias and sudden death [24]. Deletion of *Erbb2* in both atria and ventricles results in an earlier onset of cardiac pathology than the deletion of *Erbb2* solely in ventricular cardiomyocytes [65]. A requirement for Erbb2 function in the atria may persist into the adult given the increased severity of cardiomyopathy arising from deletion of *Erbb2* in cardiomyocytes of all chambers.

The mechanism of the atrial block resulting from the *Erbb2* mutation remains unclear. We speculate that the possible mechanism involves abnormalities in action potential transduction through the atrial myocardium rather than the generation of the signal by the SAN, because normal ventricular signals are observed at a regular pace independently of the presence or absence of atrial signals. The shape and duration of the action potentials present in the *l11Jus8* mutants do not appear to be altered in comparison to the signals in the control hearts. Given that Erbb2 is a transmembrane protein, it could be involved (directly or indirectly) in the restoration of the membrane potential after action potential transduction. Thus, ablation of Erbb2 activity in *l11Jus8* mutants may lead to the slowdown and eventually complete block of the membrane potential restoration and abortion of signal transduction. The hearts used in electrical mapping did not have any apparent atrial distension or necrosis, and were beating at the time of analysis. However, it remains possible that a subtle alteration in atrial cell function occurs prior to the detection of necrosis in mutant embryos, and this alteration subsequently contributes to the cardiac conduction system defects.

The embryonic lethality observed in the *l11Jus8* mutant also suggests that ERBB2 mutations may contribute to miscarriages, and the identification in the 1000 genomes dataset of multiple variations within the ERBB2 kinase domain predicted to be detrimental confirms that potentially damaging alleles are present in the human population. Additionally, our research on the *l11Jus8* mutant may inform future investigations into chemotherapy-induced cardiotoxicity associated with inhibition of ERBB2 signalling, as new anti-cancer therapeutics are being designed to target the ERBB2 kinase domain [67].

The use of a random mutagenesis approach, which prompted the characterisation of a missense mutation within the kinase domain of Erbb2, led to the discovery of a requirement for *Erbb2* in atrial electrical conduction. This role for Erbb2 had not been identified from prior studies on null or phosphorylation site mutants, demonstrating the advantages of using a random mutagenesis approach to annotate gene function. Given the dearth of research investigations into atrial development, our work demonstrates the importance of atrial function for embryonic survival, and highlights the need for additional studies on embryonic atrial function to link Erbb2 kinase domain defects to cardiac conduction system development.

## Supporting Information

Figure S1 **Series of atrial conduction maps with different thresholds. Wild type (wt):** Background noise, clearly visible at threshold value 0, 1 and 2, disappears at threshold 3, and first gap in conduction map is visible at threshold 4 (denoted by asterisk). Heterozygote **(het no gaps):** background noise disappears at threshold 2 and first gaps are visible at threshold 3 (asterisk). Heterozygote **(het gaps)** and ***l11Jus8*** mutant **(hz gaps):** gaps (asterisk) are detected at any threshold value including 0, when no noise is cut off. Maps reconstructed from E12.5 embryonic hearts.
(TIF)

Figure S2 **Electrical signals in E12.5 hearts with complete, gapped and absent maps. (A, E, I)** Whole heart view. Square denotes the area shown in **(B, D, F, H, J, L)**. **(B, F, J)** Magnified atria are shown. Black, red and blue asterisks denote the points where the electrical readings were taken. **(C, G, K)** Electrical readings at different areas of the atria shown in **(B, F, J)**. Colour of the reading corresponds to the colour of the asterisk. White arrowhead denotes the area where ventricular signal was present (not shown) but no atrial signal was detected. **(D, H, L)** Conduction maps reconstructed from the readings on **(C, G, K)**, respectively. Note the presence of clear signal in all atrium in **(B-C)** and complete conduction map on **(D)** while for atrium in **(F)**, strong signal is present in the posterior end (blue), weak signal in the central area (red) and virtually no signal in the anterior area (black). Corresponding conduction map shows significant gap in anterior and central area of the atrium **(H)**. No map **(L)** could be reconstructed from the reading where atrial signal was absent, white arrowhead in **(K)**.
(TIF)

Figure S3 **Western Blot analysis of p-ERK in** ***l11Jus8*** **hetero- and homozygous animals.** Total protein extracts from 6 E13.5 atria and ventricle (pooled separately) for each genotype were probed with Sigma activated mouse α MAP Kinase (M8159) at 1:2000 overnight at 4°, followed by a secondary incubation with Goat α Mouse-HRP (Thermo Scientific 32430) at 1∶5000 for 1 hour at room temperature. Bands were normalized against the loading control α MYH10 11-B (Covance PRB-445P).
(TIF)

Figure S4 **Comparison of the wt,** ***l11Jus8*** **hetero- and homozygous phenotypes at E12.5.** Images of internal organs within the thoracic cavity. Structures are labelled in wt images. Lu, lungs; Li, liver; Du, duodenum; Ki, kidney bud; Pa, pancreatic primordium; St, stomach. Scale bars  =  1 mm.
(TIF)

Figure S5 **Characterisation of yolk sac vessels in wt,** ***l11Jus8*** **hetero- and homozygous embryos.** Embryos within the yolk sac (A-C) show a lack of blood within the vessels in *l11Jus8* mutants. PECAM staining of yolk sac vessels (D-L) shows a progressive narrowing or regression of vessels specifically in *l11Jus8* mutant yolks sacs from E10.5 to E12.5. Genotypes and developmental stages are labelled on the figure. Scale bars: 2 mm in (A–C), 1 mm in ((D–L).
(TIF)

Figure S6 **Characterisation of the atrial wall of the wt,** ***l11Jus8*** **hetero- and homozygous hearts. (A**–**C)** *In situ* hybridisation for cardiomyocyte marker *Myl4*. **(D**–**F)** α-SMA immunostaining. **(G**–**I)** Laminin immunostaining. **(J**–**L)** Fibronectin immunostaining. **(M**–**O)** Phospho-histone H3 immunostaining. Arrowheads point to the positively-stained cells. **(P**–**R)** DAPI labelling of the nuclei. En, endocardium; Ep, epicardium; My, myocardium. Arrowheads in **(R)** denote the areas containing cells with fragmented nuclei. Scale bars: 1 mm in **(A**–**C)**, 100 µm in **(D**–**L, P**–**R)**, 40 µm in **(M**–**O)**. Developmental stage of embryos: (A–C) E10.5, (D–L) E12.5, (M–R) E11.5.
(TIF)

Figure S7 **Series of conduction maps reconstructed for the E12.5 heart with atrial block.** No map could be reconstructed for the missing atrial signal **(A4)** while corresponding ventricular signal was present and complete ventricular map was reconstructed **(V4)**. Notably, all atrial map reconstructed from present atrial signals **(A1**–**A3, A5)** contain significant gaps while corresponding ventricular maps are complete **(V1**–**V3, V5)**.
(TIF)

Figure S8 **Expression of Connexins 43 and 40 in E12.5 hetero- and homozygous** ***l11Jus8*** **hearts. (A**–**B) and (C**–**D)** Sagittal sections of E12.5 hetero- and homozygous *l11Jus8* hearts stained with Cx43 and Cx40 antibodies, respectively. Atr, atrium; Ven, ventricle. Arrow heads point to the autofluorescent erythrocytes.
(TIF)

Table S1 **List of sequenced positions on chr11:69**–**103 Mb in** ***l11Jus8*** **mouse.**
(DOCX)

Table S2 **Analysis of lethality in recombinant animals.**
(DOCX)

Table S3 **Viability of the** ***l11Jus8*** **embryos.**
(DOCX)
