# Supplementary figures and images for: Erbb2 Is Required for Cardiac Atrial Electrical Activity during Development
Source: PLoS One. 2014 Sep 30;9(9):e107041. doi: 10.1371/journal.pone.0107041 (PMC4182046; doi:10.1371/journal.pone.0107041)

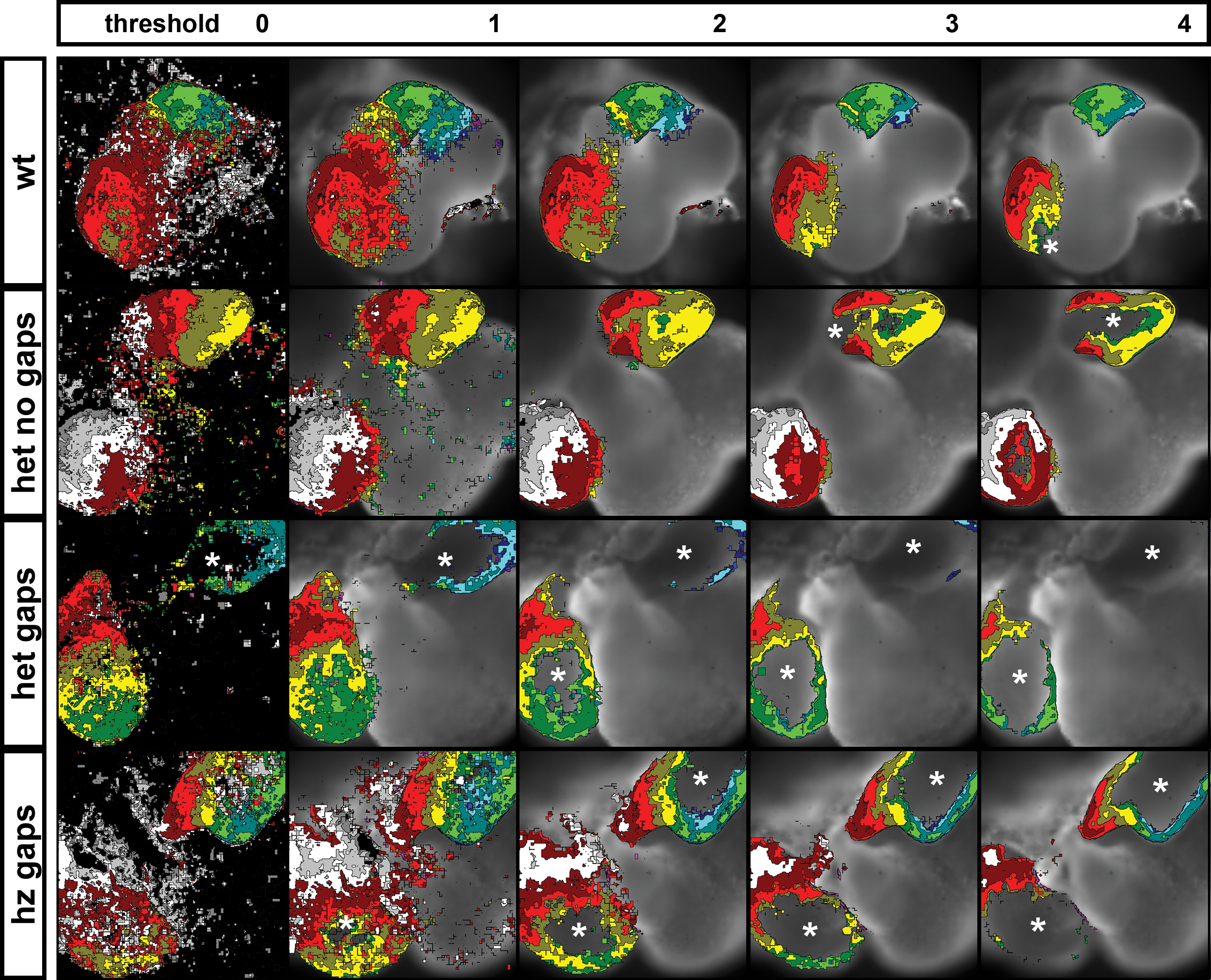

Supplement: Figure S1 — Series of atrial conduction maps with different thresholds. Wild type (wt): Background noise, clearly visible at threshold value 0, 1 and 2, disappears at threshold 3, and first gap in conduction map is visible at threshold 4 (denoted by asterisk). Heterozygote (het no gaps): background noise disappears at threshold 2 and first gaps are visible at threshold 3 (asterisk). Heterozygote (het gaps) and l11Jus8 mutant (hz gaps): gaps (asterisk) are detected at any threshold value including 0, when no noise is cut off. Maps reconstructed from E12.5 embryonic hearts. (TIF) [file pone.0107041.s001.tif]

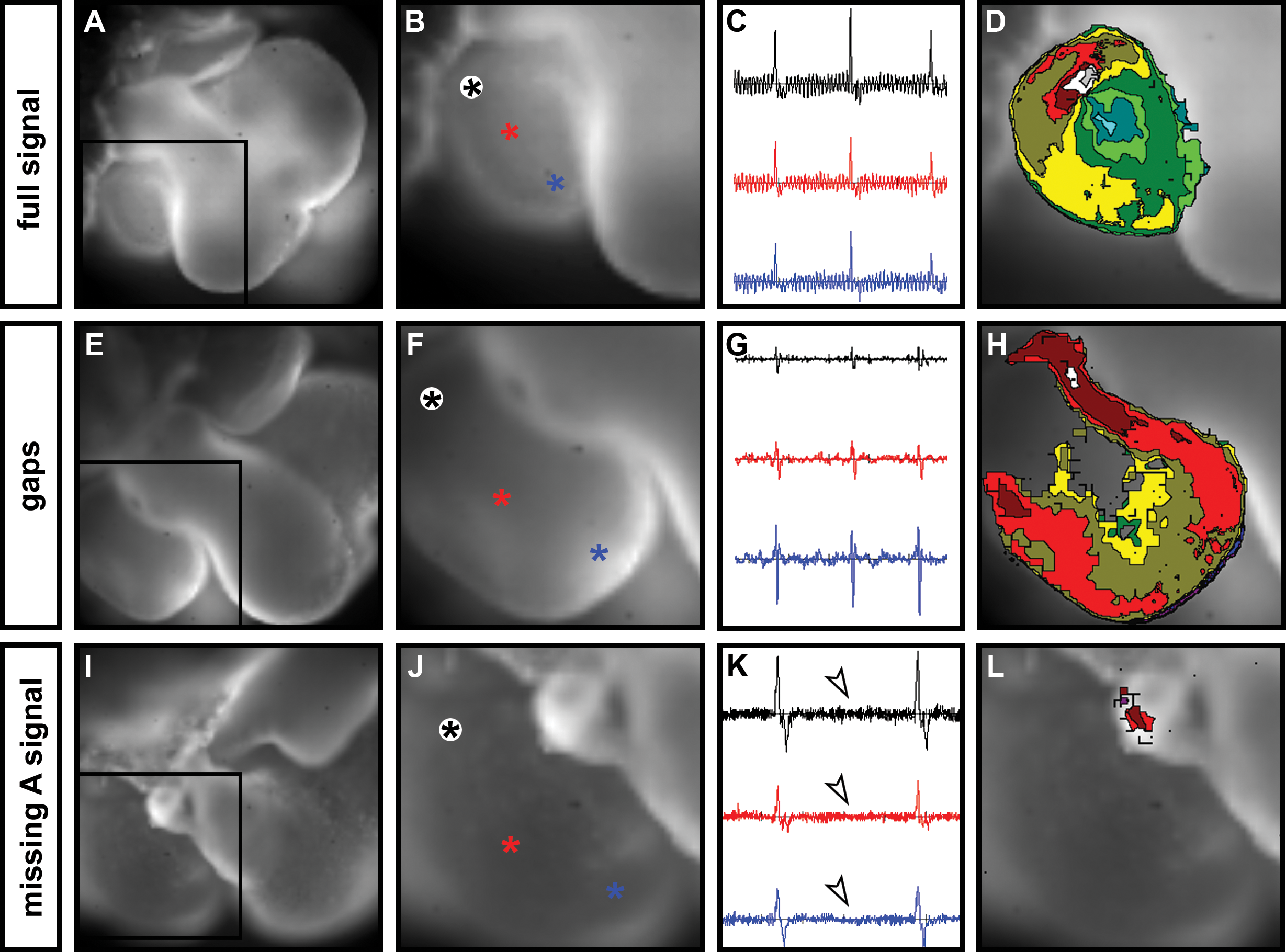

Supplement: Figure S2 — Electrical signals in E12.5 hearts with complete, gapped and absent maps. (A, E, I) Whole heart view. Square denotes the area shown in (B, D, F, H, J, L). (B, F, J) Magnified atria are shown. Black, red and blue asterisks denote the points where the electrical readings were taken. (C, G, K) Electrical readings at different areas of the atria shown in (B, F, J). Colour of the reading corresponds to the colour of the asterisk. White arrowhead denotes the area where ventricular signal was present (not shown) but no atrial signal was detected. (D, H, L) Conduction maps reconstructed from the readings on (C, G, K), respectively. Note the presence of clear signal in all atrium in (B-C) and complete conduction map on (D) while for atrium in (F), strong signal is present in the posterior end (blue), weak signal in the central area (red) and virtually no signal in the anterior area (black). Corresponding conduction map shows significant gap in anterior and central area of the atrium (H). No map (L) could be reconstructed from the reading where atrial signal was absent, white arrowhead in (K). (TIF) [file pone.0107041.s002.tif]

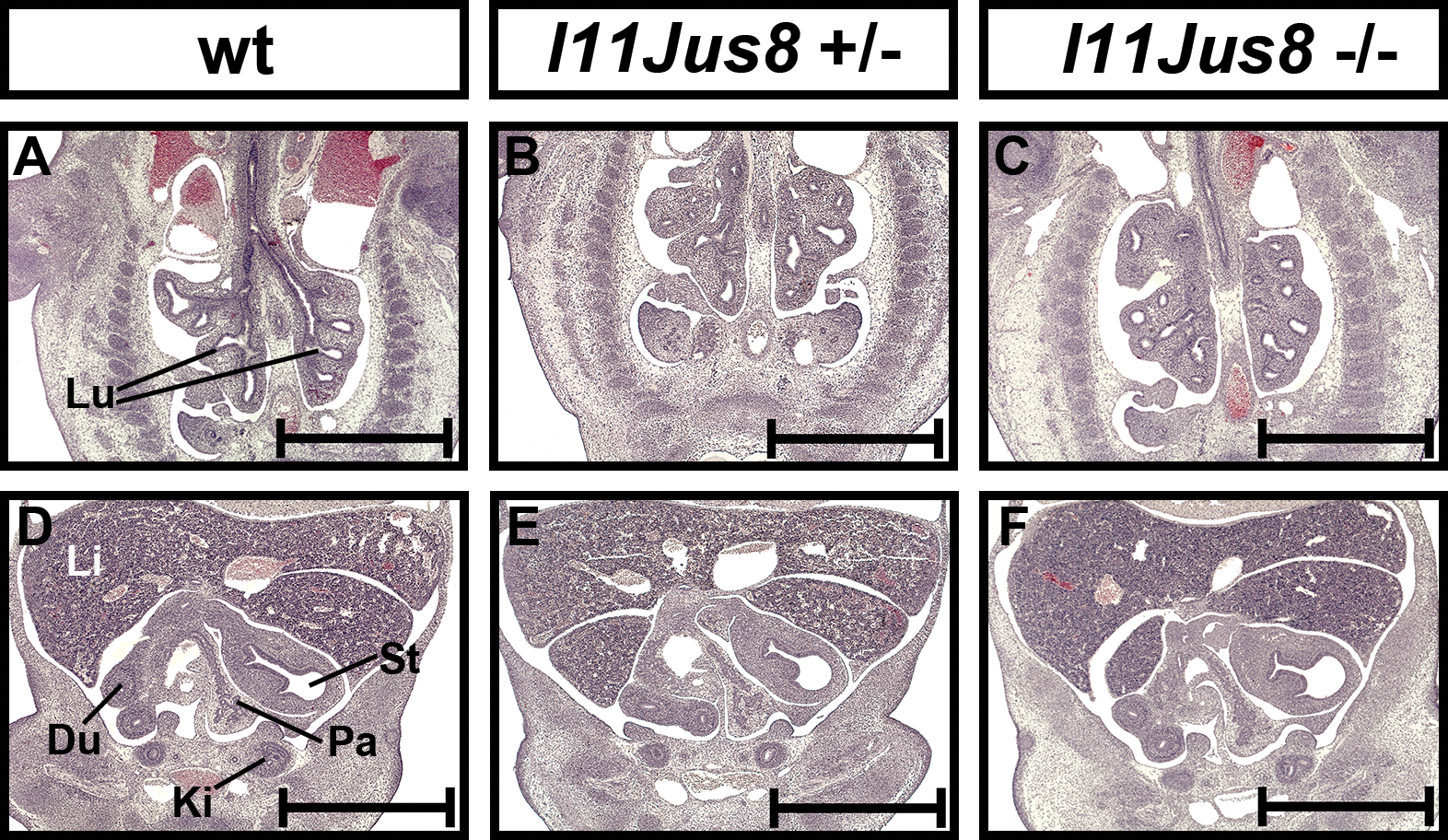

Supplement: Figure S4 — Comparison of the wt, l11Jus8 hetero- and homozygous phenotypes at E12.5. Images of internal organs within the thoracic cavity. Structures are labelled in wt images. Lu, lungs; Li, liver; Du, duodenum; Ki, kidney bud; Pa, pancreatic primordium; St, stomach. Scale bars = 1 mm. (TIF) [file pone.0107041.s004.tif]

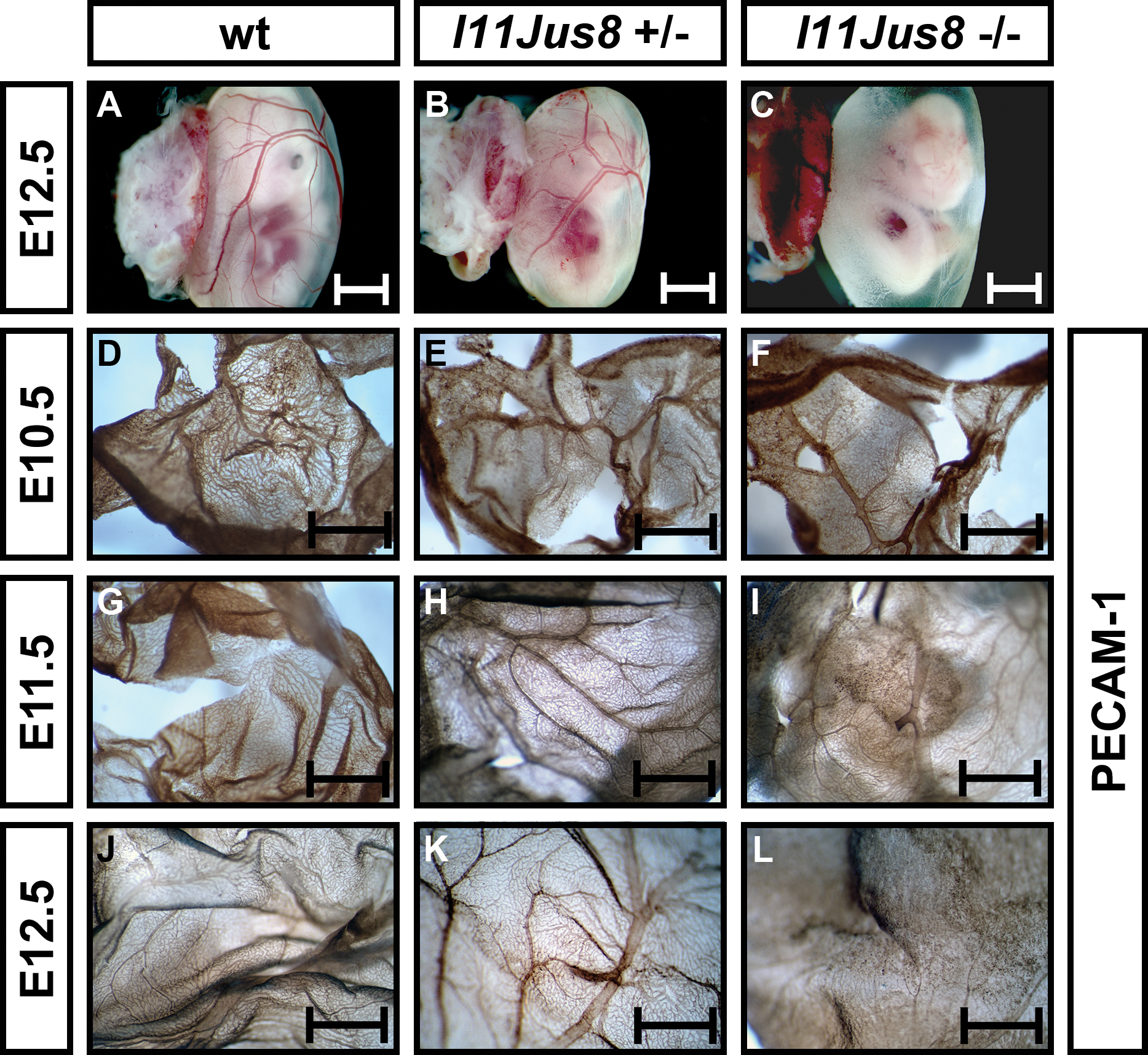

Supplement: Figure S5 — Characterisation of yolk sac vessels in wt, l11Jus8 hetero- and homozygous embryos. Embryos within the yolk sac (A-C) show a lack of blood within the vessels in l11Jus8 mutants. PECAM staining of yolk sac vessels (D-L) shows a progressive narrowing or regression of vessels specifically in l11Jus8 mutant yolks sacs from E10.5 to E12.5. Genotypes and developmental stages are labelled on the figure. Scale bars: 2 mm in (A–C), 1 mm in ((D–L). (TIF) [file pone.0107041.s005.tif]

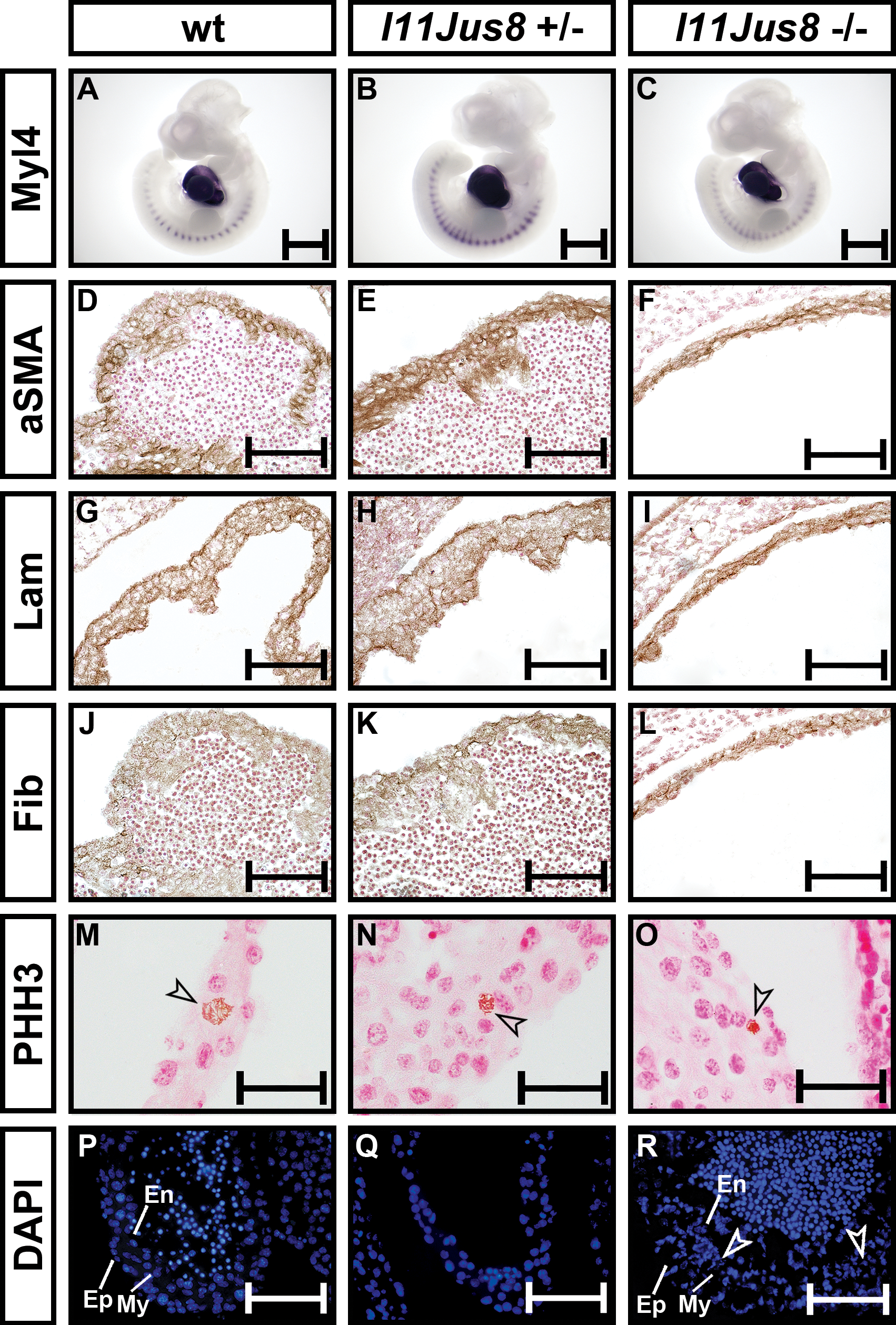

Supplement: Figure S6 — Characterisation of the atrial wall of the wt, l11Jus8 hetero- and homozygous hearts. (A–C) In situ hybridisation for cardiomyocyte marker Myl4. (D–F) α-SMA immunostaining. (G–I) Laminin immunostaining. (J–L) Fibronectin immunostaining. (M–O) Phospho-histone H3 immunostaining. Arrowheads point to the positively-stained cells. (P–R) DAPI labelling of the nuclei. En, endocardium; Ep, epicardium; My, myocardium. Arrowheads in (R) denote the areas containing cells with fragmented nuclei. Scale bars: 1 mm in (A–C), 100 µm in (D–L, P–R), 40 µm in (M–O). Developmental stage of embryos: (A–C) E10.5, (D–L) E12.5, (M–R) E11.5. (TIF) [file pone.0107041.s006.tif]

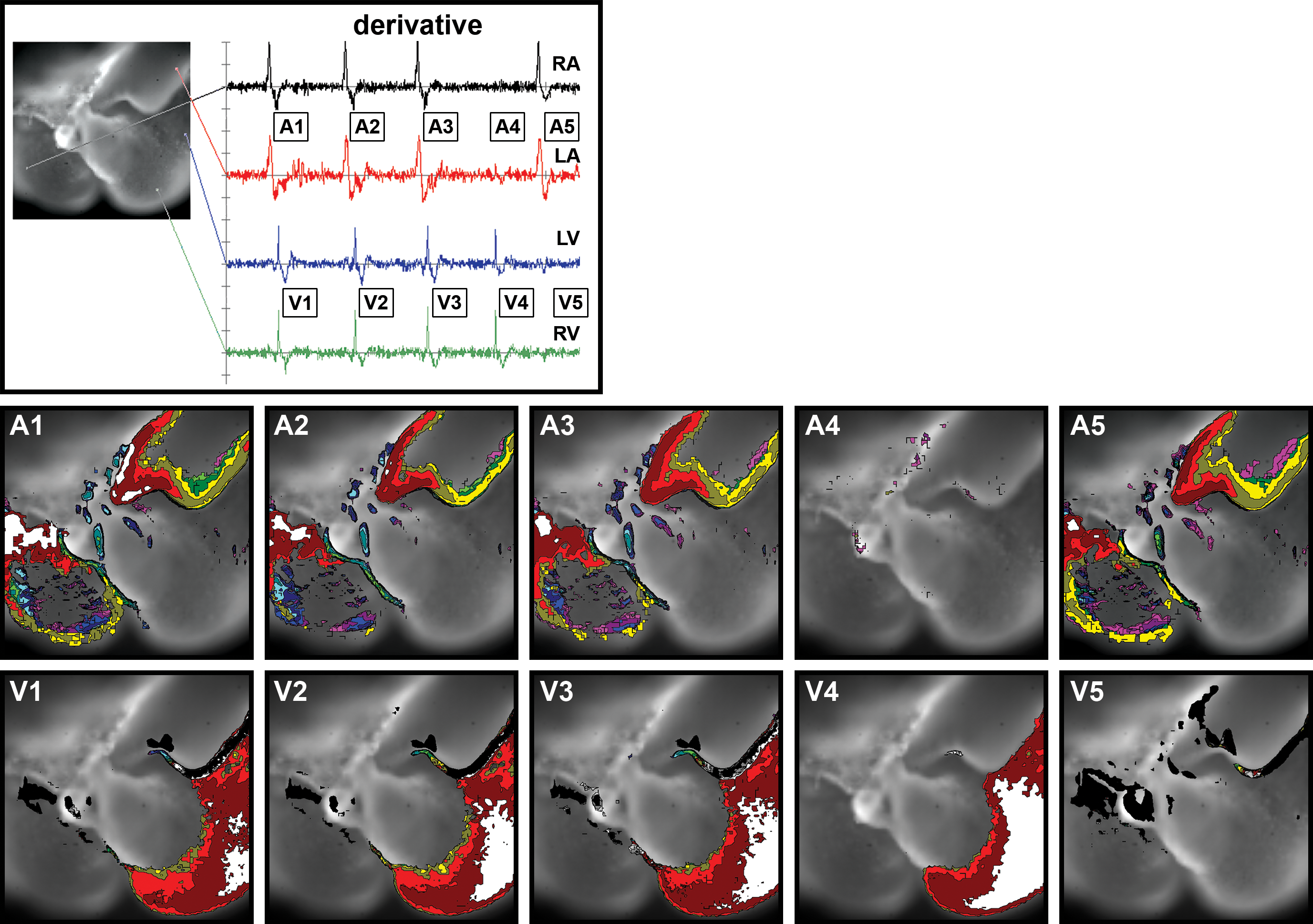

Supplement: Figure S7 — Series of conduction maps reconstructed for the E12.5 heart with atrial block. No map could be reconstructed for the missing atrial signal (A4) while corresponding ventricular signal was present and complete ventricular map was reconstructed (V4). Notably, all atrial map reconstructed from present atrial signals (A1–A3, A5) contain significant gaps while corresponding ventricular maps are complete (V1–V3, V5). (TIF) [file pone.0107041.s007.tif]

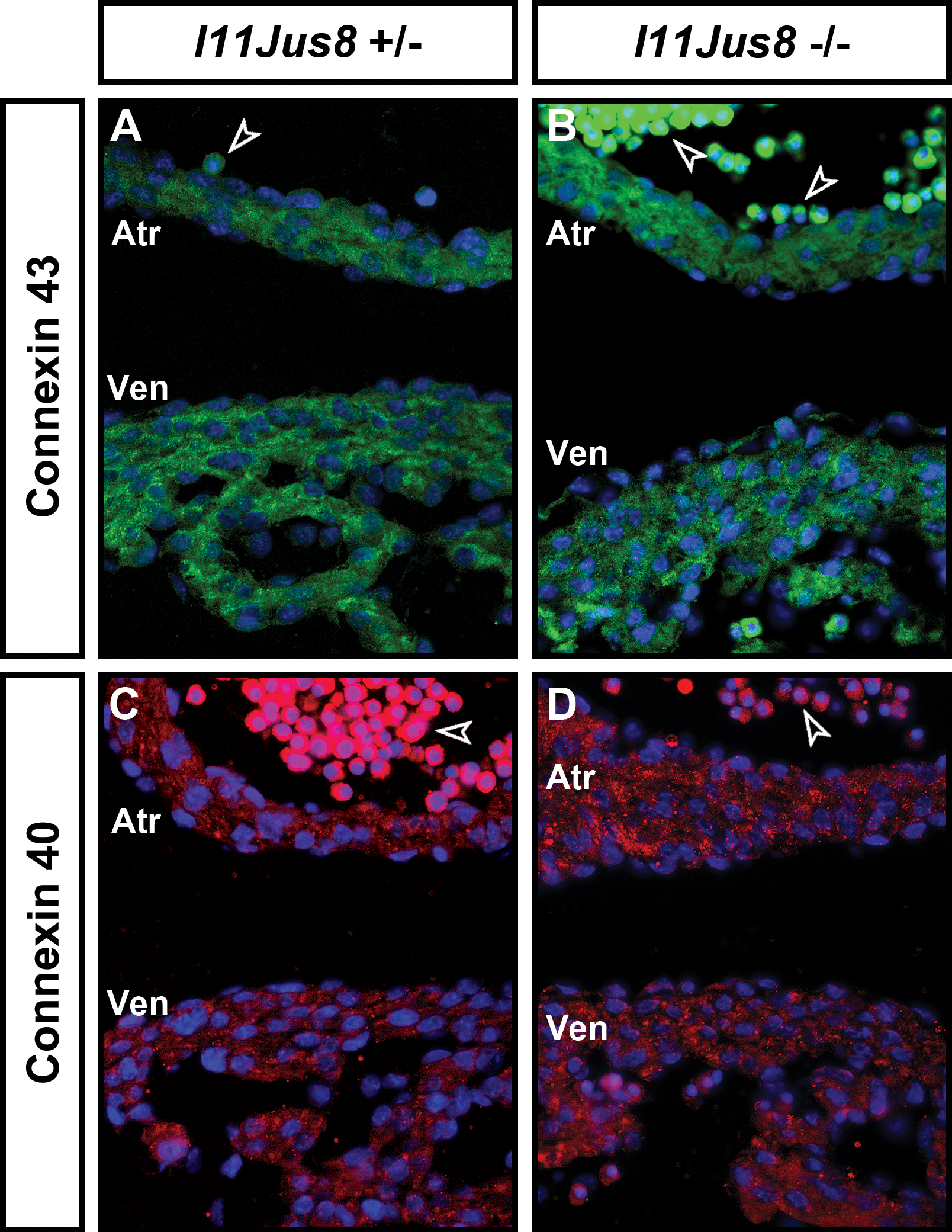

Supplement: Figure S8 — Expression of Connexins 43 and 40 in E12.5 hetero- and homozygous l11Jus8 hearts. (A–B) and (C–D) Sagittal sections of E12.5 hetero- and homozygous l11Jus8 hearts stained with Cx43 and Cx40 antibodies, respectively. Atr, atrium; Ven, ventricle. Arrow heads point to the autofluorescent erythrocytes. (TIF) [file pone.0107041.s008.tif]
