# Supplementary material for: Erbb2 Is Required for Cardiac Atrial Electrical Activity during Development
Source: PLoS One. 2014 Sep 30;9(9):e107041. doi: 10.1371/journal.pone.0107041 (PMC4182046; doi:10.1371/journal.pone.0107041)
Supplement: Table S1 — List of sequenced positions on chr11:69–103 Mb in l11Jus8 mouse. (DOCX) [file pone.0107041.s009.docx]

**Table S1. List of sequenced positions on chr11:69-103 Mb in *l11Jus8* mouse.**

| **position** | **base change** | **annotation** | **status** |
| --- | --- | --- | --- |
| 70023993 | T/C | Dlg4 | false positive |
| 72213810 | T/C | Med31 | confirmed |
| 73395083 | T/G | Olfr1 | false positive |
| 76211485 | C/T | Gemin4 | false positive |
| 79447501 | T/A | NF1 | confirmed |
| 83985389 | T/A | Synrg | false positive |
| 85837942 | G/T | Tbx2 | false positive |
| 97022939 | C/A | Novel processed transcript | false positive |
| 98230132 | G/A | Cdk12 | false positive |
| 98433986 | T/G | Erbb2 | confirmed |
| 100117606 | C/T | Krt13 | false positive |
| 102863803 | A/T | lincRNA | false positive |
| 103347994 | C/T | novel AS-RNA | false positive |
