# Supplementary material for: Erbb2 Is Required for Cardiac Atrial Electrical Activity during Development
Source: PLoS One. 2014 Sep 30;9(9):e107041. doi: 10.1371/journal.pone.0107041 (PMC4182046; doi:10.1371/journal.pone.0107041)
Supplement: Table S2 — Analysis of lethality in recombinant animals. (DOCX) [file pone.0107041.s010.docx]

**Table S2. Analysis of lethality in recombinant animals.**

| **Recombinant** | **# C57BL/6 viable offspring** | **total offspring** | **deviation from expected**  **(p-value)** |
| --- | --- | --- | --- |
| rec1 | 6 | 24 | 1 |
| rec2 | 3 | 21 | 0.256 |
| rec3 | 1 | 8 | 0.414 |
| rec4 | 0 | 23 | 0.0056 |
| rec5 | 0 | 11 | 0.055 |
