# Supplementary material for: Erbb2 Is Required for Cardiac Atrial Electrical Activity during Development
Source: PLoS One. 2014 Sep 30;9(9):e107041. doi: 10.1371/journal.pone.0107041 (PMC4182046; doi:10.1371/journal.pone.0107041)
Supplement: Table S3 — Viability of the l11Jus8 embryos. (DOCX) [file pone.0107041.s011.docx]

**Table S3. Viability of the *l11Jus8* embryos.**

| **Developmental Time Point** | **Wild Type** | **Heterozygote** | ***l11Jus8*** |
| --- | --- | --- | --- |
| E10.5 | 6 | 23 | 12 |
| E11.5 | 9 | 14 | 10 of 12 viable |
| E12.5 | 5 | 14 | 4 of 12 viable |
